# Pulmonary Disease Secondary to Reflux Mimicking Interstitial Pneumonia in Systemic Sclerosis: Case Report and Literature Review

**DOI:** 10.1155/2016/5926327

**Published:** 2016-01-14

**Authors:** Ricardo Azêdo de Luca Montes, Nathalia Mazolli Veiga, Pedro Gemal Lanzieri, Luis Otávio Cardoso Mocarzel

**Affiliations:** ^1^Department of Rheumatology, Hospital Universitário Antônio Pedro (HUAP), Universidade Federal Fluminense (UFF), Rua Marquês de Paraná 303, 7° Andar, Centro, 24033-900 Niterói, RJ, Brazil; ^2^Department of Internal Medicine, Hospital Universitário Antônio Pedro (HUAP), Universidade Federal Fluminense (UFF), Rua Marquês de Paraná 303, 7° Andar, Centro, 24033-900 Niterói, RJ, Brazil

## Abstract

Systemic sclerosis is a complex disease due to the variety of clinical presentations, often superimposed on other conditions, related or not to the connective tissue. We report a 43-year-old Brazilian woman with limited systemic sclerosis and pulmonary symptoms secondary to gastroesophageal reflux disease, with a clinical presentation similar to a diffuse interstitial lung disease. Because of the frequency of interstitial lung injury due to systemic sclerosis, this was an important differential diagnosis, which could be excluded after optimized treatment of reflux disease, with clinical and radiological improvement. Clinical management of patients with collagen diseases requires clinician skills to identify the natural history and understand its nuances. This is a common situation in clinical practice, but with a few discussions in international literature.

## 1. Introduction

Systemic sclerosis (SSc) is a syndrome with variable presentation and that can present superimposed on other diffuse connective tissue diseases such as Systemic Lupus Erythematosus (4% of cases), polymyositis, Sjögren's Syndrome, and Rheumatoid Arthritis [1].

On its usual presentation and progression, esophagus is the most affected internal organ in ES, with 50% to 90% of patients with gastrointestinal complaints [1]. Diffuse interstitial lung disease (ILD) is the leading cause of death in the progressive ES with histologic pattern of nonspecific interstitial pneumonia, whose response to immunosuppressive treatment is often unsatisfactory [1–3]. In the case of localized systemic sclerosis, there is a predominance of cutaneous signs, followed in frequency by esophageal dysfunction [3]. The high morbidity of this clinical condition is related to restrictive changes of joint mobility by skin thickening and to primary pulmonary arterial hypertension [4].

There are few reports in the literature that relate the influence of treatment of reflux disease (GERD) to the progression of ILD [2, 3].

## 2. Case Presentation

A 43-year-old Black woman, born in Rio de Janeiro, southeast of Brazil, presents to the outpatient clinic with a six-month history of shortness of breath and dyspnea on exertion and recently started unproductive cough and intermittent fever. She had no other respiratory or cardiovascular complaints. During that period, she has been treated three times empirically for community acquired pneumonia, with no improvement.

She has been diagnosed with Systemic Lupus Erythematosus four years earlier by the following criteria: facial rash, oral ulcer, hemolytic anemia, serositis, and positive antinuclear antibodies titers at 1 : 1280. She was on mycophenolate mofetil 1 g bid, prednisone 5 mg qd, hydroxychloroquine 400 mg qd, and sunscreen.

Her physical examination was remarkable for a heart rate of 120 beats per minute with no fever, a respiratory rate of 28 breaths per minute, with a slight respiratory effort, and peripheral saturation of 96% and axillary temperature of 36°C. She had dyspnea on moderate efforts and dysphonia. On lung examination, expandability was reduced and there were crackles bilaterally. Cardiovascular system showed an A2 component of the second heart sound louder than P2. Skin thickening was evident on forearms and there was hyperpigmentation with telangiectasia on the face and on the fingertips. Hand examination was positive for sclerodactyly and for Raynaud's phenomenon.

Direct laryngoscopy showed thickening of external vocal cords, hyperemia, and interarytenoid edema.

Autoantibody panel was positive for antinuclear antibodies with titers 1/1280 (fine speckled) and anticentromere antibody and dosage for anti-Scl 70 greater than fifteen times the normal range.

For cardiopulmonary study, a spirometry showed a severe restrictive pattern with negative results on bronchodilator test, moderately reduced carbon monoxide diffusion, and no sign of obstructive airway disease. High resolution tomography of the chest identified pulmonary areas of ground-glass infiltrates in the upper left lobe and, even more intense, in the lower lobes, showing small foci of condensation in the posterior regions. Doppler echocardiography showed diastolic dysfunction grade I, with normal pulmonary artery pressure, and the level of serum brain natriuretic peptide was normal.

For gastrointestinal evaluation, an upper endoscopy was done, and the result was reflux esophagitis graded C on Los Angeles classification and chronic active gastritis. Esophageal manometry showed a motor disorder with marked esophageal dysmotility, mainly on the lower two-thirds of the esophagus. 24-hour pH-impedance monitoring was not performed because it was not available at that time.

At that time, it was decided to start treatment for gastroesophageal reflux disease (GERD)—although there were no typical reflux symptoms—and to perform a skin biopsy. A distal area of her left forearm was chosen, and histopathology suggested overlap lupus and limited systemic sclerosis overlap.

After optimization of treatment with full-dose proton pump inhibitor (80 mg/daily) and prokinetic (dopamine antagonist) medications for 8 weeks, the patient regressed symptoms. She showed an improvement in dyspnea, complete resolution of hoarseness, and significant improvement in the radiological lung image. There was a reduction of areas with ground-glass infiltrates, with no signs of parenchymal consolidation or new interstitial lesions.

Due to the improvement of pulmonary lesions shown by computed tomography, including clearance of the lesion in the left upper lobe, it was decided to maintain the optimal treatment of GERD with respiratory sustained clinical improvement after four months.

**Figure 1:**
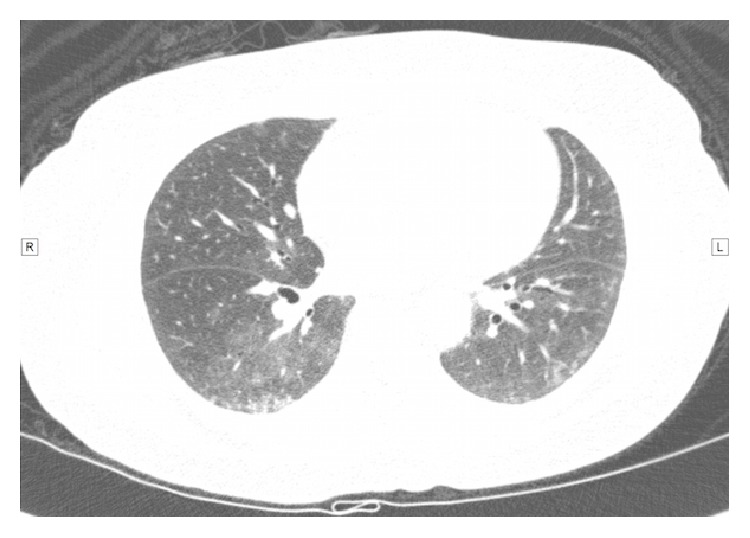
Chest tomography scan previous to treatment. T8 level with 10-millimeter sections. Pulmonary interstitium is affected bilaterally, with bronchiectasis on the lower lobes. Subpleural lines are preserved, suggesting unspecific interstitial pneumonia.

**Figure 2:**
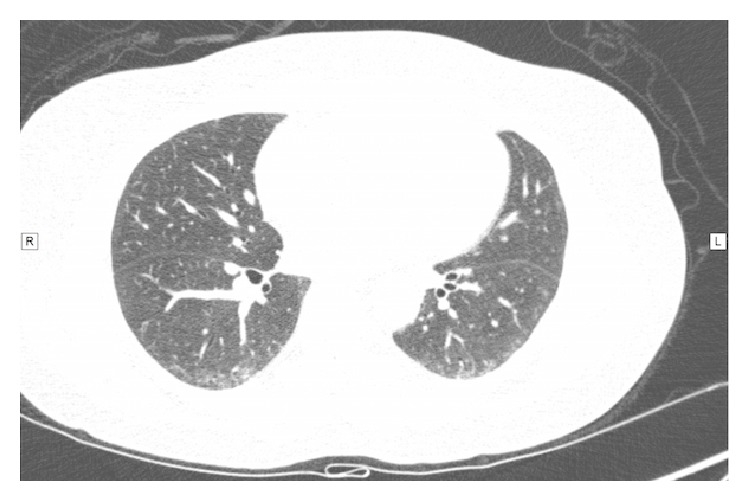
Chest tomography scan after two weeks of treatment. Images on same level and resolution. Note the improvement on the ground-glass pattern on lower lobes.

Figures 1 and 2 show the pattern of high resolution computerized tomography, before and after treatment for GERD.

## 3. Discussion

The development of lung disease in systemic sclerosis is associated with the anti-Scl 70 antibody and correlates with worsening of lung function in disease progression, measured by forced vital capacity. In the mentioned case, there was no worsening of lung function.

The prevalence of diffuse interstitial lung disease (DIP) in patients with systemic sclerosis is reported in up to 70% of cases, mostly in patients with diffuse SSc [1]. Anti-Scl 70 is a factor related to this condition and already identified as an independent predictor of poor prognosis [5]. Pulmonary involvements in overlapping syndromes are less frequent, with national statistics around 50% of cases [6].

Although they are not commercially available, studies suggest that the positive anti-Th/To and Anti-Ku may be useful markers for the DIP associated with SSc [7]. The assessment of these markers could decrease the diagnostic doubts as reported cases [7, 8].

As the chest image was not able to rule out the possibility of microaspiration with infectious inflammation in the posterior regions of the lower lobes, and esophageal ecstasies, it was decided to investigate esophageal disease.

Disorders of the esophagus engines are described as a contributing factor for PID in patients with SSc [3]. Esophageal changes with decreased peristalsis and lower esophageal sphincter pressure may induce progressive histological damage due to microaspiration of gastric contents into the respiratory tract [3, 5, 8, 9]. In the mentioned case, evaluation of esophageal and lung function by manometry and respiratory function test, respectively, was not able to establish a relation between the degree of involvement of the esophagus and lung in patients with scleroderma [10].

The establishment of a causal relationship between esophagus and lung involvement in SSc is important since the antireflux treatment can reduce morbidity related to lung involvement in patients with SSc [10].

In this report, the patient had an esophageal dysmotility disorder, markedly in the distal part, while in the literature the prevalence in patients with diffuse connective tissue diseases and lung involvement is mainly hypomotility disorders or absence of peristalsis. The average age at which this diagnosis is often made is 51, relatively close to said case (43 years) [9, 10]. In our case, the improvement of symptoms and radiological changes only due to the optimal treatment for GERD discourages the diagnosis of PID, in which case the response to treatment depends on immunosuppression, with long-term variance. The presence of hoarseness was a clinical indicator of the possibility of GERD with atypical manifestations. The temporary return of symptoms that coincided with the suspension of prokinetic medications confirmed the importance of esophageal component in the pathophysiology of pulmonary involvement. Regardless of these facts, studies have proved the existence of important acid and nonacid reflux, also in patients with idiopathic pulmonary fibrosis [10].

Faced with a suspected interstitial lung disease in a patient with scleroderma, always evaluate pulmonary topography and consider (1) activity of the scleroderma disease; (2) pulmonary disease secondary to GERD; (3) GERD aggravating the LID of scleroderma.
